# How Would Economic Development Influence Carbon Productivity? A Case from Hubei in China

**DOI:** 10.3390/ijerph15081730

**Published:** 2018-08-12

**Authors:** Yiwei Wang, Shuwang Yang, Canmian Liu, Shiying Li

**Affiliations:** 1 School of Economic Management, China University of Geosciences, Wuhan 430074, China; yiweifiona@hotmail.com (Y.W.); yswang998@163.com (S.Y.); 2 Center for Research of Economics & Environment, China University of Geosciences, Wuhan 430074, China; 3 Key Laboratory of Strategic Studies, Ministry of Land and Resources, Wuhan 430074, China; 4 Business School, Sichuan University, Chengdu 610065, China; 2016225025022@stu.scu.edu.cn; 5 School of Public Administration, Sichuan University, Chengdu 610064, China

**Keywords:** economic growth, carbon productivity, smooth transition regression model, Markov regime switching model

## Abstract

Carbon productivity, defined as the gross domestic product (GDP) per unit of CO_2_ emissions, has been used by provincial governments in China as in indicator for effort and effect in addressing climate-change problems. The aggregate impact of economic growth on carbon productivity is complex and worthy of extensive investigation to design effective environmental and economic policies. Based on a novel combination of the smooth transition regression model and the Markov regime-switching regression model, this paper analyzes time series data on carbon productivity and economic growth from Hubei Province in China. The results show that the influence of economic growth on carbon productivity is highly nonlinear. In general, economic growth has a positive impact on improving carbon productivity. From a longitudinal perspective, this nonlinear positive impact is further divided into three stages, transiting from a high regime to a low regime and then back to a high regime. The high regime stage, in which economic growth has stronger positive influence on enhancing carbon productivity, is expected to last for considerably longer time than the low regime stage. It is more probable for a low regime stage to transit to a high regime. Once the relation of carbon productivity and economic growth enters the high regime status it becomes relatively stable there. If the government aims to achieve higher carbon productivity, it is helpful to encourage stronger economic development. However, simply enhancing carbon productivity is not enough for curbing carbon emissions, especially for fast growing economies.

## 1. Introduction

In order to achieve the goal of stopping global warming at 2 °C above pre-industrial levels, anthropogenic greenhouse gas emissions need to be carefully controlled [1]. On the other hand, economic growth, while it improves social welfare, continuously generates huge amounts of greenhouse gas emissions. How to coordinate economic growth and greenhouse gas abatement has become an important problem for governments, especially in developing countries. Since the majority of greenhouse gas emissions is carbon dioxide (CO_2_), in the rest of this article, the focus is on CO_2_ emissions.

China is now the world’s largest producer of CO_2_ emissions, and thereby bears huge responsibility for curbing its emissions. To slow down production of CO_2_ emissions while sustaining economic growth, the Chinese government seeks to increase carbon productivity, which is defined by the gross domestic product (GDP) per unit of CO_2_ emissions. Carbon productivity has been the key performance indicator (KPI) for evaluating provincial governments’ performance since 2014. According to the data from the World Bank, the carbon productivity of China in 2014 is US$810 per ton of CO_2_ emission, which is significantly lower than the world’s average carbon productivity of US$2035 per ton of CO_2_ emission. Although China has made significant progresses in controlling its emissions, there is still much room for improvement. The Chinese government has formally committed to reduce its carbon intensity (the reciprocal of carbon productivity) by 60–65% from the 2005 level by 2030. In China’s fast developing economy, the relationship between economic growth and carbon productivity is complex and may change during different time periods. Fulfilling the Chinese government’s pledge of carbon emissions requires a deeper understanding of that relationship.

Within a wider scope, the relationship between economic growth and environmental impact has been extensively studied. There are two streams of literature on this issue. The first stream focuses on the environmental Kuznets curve (EKC) theory. In the seminal work of Grossman and Krueger [2], the relationship between economic growth and environmental quality is described by an inverted U-shaped curve. This inverted U-shaped relationship is due to the interactions between resource utilization, technology improvement and economic structure changes [3,4]. However, there are debates about other shapes of the EKC curves [5,6,7]. Some scholars even think the curve does not exist [8,9]. The other stream is the analyses on factors influencing the resources and environment based on the stochastic impacts by regression on population, affluence and technology (STIRPAT) model. The STIRPAT model generally considers population, affluence level and technology as the main factors affecting a region’s environment [10,11,12].

There is considerable literature specifically focused on carbon emissions rather than general environmental impacts. For example, Holtz-Eakin and Selden [13] studied the relationship between economic growth and carbon emissions using panel data of 130 countries. Wang et al. [14] investigated the causes and regime transitions in the dynamics of carbon emissions in China. The nonlinear relationship between carbon emissions and other factors are modeled by quadratic functions or transition functions. Many of these studies consider carbon emission per capita as the dependent variable, whereas the Chinese government chooses carbon productivity in its commitment. This is probably because China’s carbon emission per capita is still very low because of its huge population, and thereby does not directly reflect the quality of low-carbon economic growth, so the rate of carbon productivity growth is considered to better weigh the efforts to address climate change and the corresponding effect [15]. The concept of carbon productivity can be further generalized to measure the efficiency of low-carbon economies [16].

The studies on carbon productivity mainly take two perspectives. The first perspective compares carbon productivity across different times, different industries and different regions [17,18,19,20], trying to find underlying patterns. The results indicate that carbon productivity is gradually increasing but is changing with different patterns among industries and regions. The differences in carbon productivity under various scenarios could be significant, thus requiring comprehensive investigations before effective control. The second perspective investigates the factors influencing carbon productivity. The main factors include industry structure, energy consumption structure, technology level, macroeconomic policies and so forth [21,22,23,24]. However, most of the analyses on carbon productivity are based on linear models for simplification. Since the relationship between economic growth and carbon productivity may be complex and unstable, it is worthwhile to further explore the nonlinear and dynamic characteristics of this relationship.

In this paper, we choose Hubei Province in China as a case, to undertake an in depth investigation on the variational relationship between economic growth and carbon productivity. There are considerable variations in Hubei’s economic growth across different periods. Its GDP used to rank in the top 10 among China’s provinces in the 1980s, then the ranking dropped, but in recent years the GDP ranking has been climbing quickly again. Hubei has done a lot of work on improving its carbon productivity. Among the seven regional carbon trade markets in China, the Hubei carbon exchange is the largest one, accounting for 71.4% of the total trade volume in China in 2016. According to the plan of National Development and Reform Committee of China, the national carbon exchange will be established in Hubei in the near future. Our study on Hubei’s experience could provide insights for other regions seeking to improve carbon productivity. By analyzing the time series data in Hubei, we attempt to answer the following research questions: Are there any structural changes in the relationship between economic growth and carbon productivity in Hubei? What are the characteristics of the structural changes? What is the future trend for carbon productivity?

We find that no matter whether developing fast or slow, the economic growth in Hubei has a positive nonlinear impact on increasing carbon productivity. This impact can be divided into three stages, transiting from a high regime to a low regime, and then back to the high regime. The high regime, in which economic growth has a stronger positive impact on enhancing carbon productivity, is expected to last for significantly longer time than the low regime. It is more probable for a low regime to transit to a high regime. Therefore, once the relationship of carbon productivity and economic growth enters a high regime status, it becomes relatively stable there. Carbon productivity and economic growth are in a sense complements that change in the same direction when policies change. Improving carbon productivity does not necessarily hurt economic growth, but carbon productivity alone is not enough to be an indicator for curbing the total carbon emissions. The main findings provide insights for policy makers to design economic and environmental policies.

The rest of the paper is organized as follows: Section 2 describes the model and data for analysis. Section 3 presents the empirical results. Section 4 makes further discussions on the results. Section 5 summarizes the findings and concludes the paper.

## 2. Model and Data

### 2.1. Model Description

This paper analyzes the relationship between economic growth and carbon productivity using two different but related models, the smooth transition regression (STR) model and the Markov regime-switching regression (MRS) model. Both are typical nonlinear models. Each model has its own strengths. The STR model is capable of capturing the cause, the time and the form of regime transitions, but is not suitable for prediction. The MRS model can calculate the probability of variables transiting from one regime to others, thus can be used for prediction, but the MRS model is weak in capturing the causes of regime transitions. Furthermore, the MRS model requires strong assumptions such as instant transition and Markov property. These two models are complementary in their pros and cons, thus they together can provide a comprehensive understanding of the time, form and future trends for the changing relationship between economic growth and carbon productivity in Hubei. The two models are briefly introduced as follows.

#### 2.1.1. Smooth Transition Regression Model

The STR model was proposed by Bacon and Watts [25]. The model is widely used in describing structural changes in time series regressions. In reality, structural change of variables usually does not happen instantly, but is a continuous, gradually changing smooth process following certain patterns. The STR model is very suitable in capturing such transition processes.

The general form of a two-regimes STR model is:

(1) $$y_{t}=\Phi^{\prime }z_{t}\times \left(1-G\left(s_{t},\gamma ,c\right)\right)+\left(\theta^{\prime }z_{t}\right)\times G\left(s_{t},\gamma ,c\right)+\varepsilon_{t},$$

in which $y_{t}$ is the dependent variable, $z_{t}=\left(w_{t}^{\prime },x_{t}^{\prime }\right)$ is the independent variable. $w_{t}={\left(1,y_{t-1},\ldots ,y_{t-p}\right)}^{\prime }$ and $x_{t}={\left(x_{1t},\ldots ,x_{kt}\right)}^{\prime }$ are endogenous and exogenous variables respectively. $\Phi ={\left(\Phi_{0},\Phi_{1},\ldots ,\Phi_{m}\right)}^{\prime }$ and $\theta ={\left(\theta_{0},\theta_{1}^{\prime }\right)}^{\prime }={\left(\theta_{0},\theta_{1},\ldots ,\theta_{m}\right)}^{\prime }$ are the coefficient matrices. $\varepsilon_{t}$ satisfies conditions including $iid\left(0,\sigma^{2}\right)$, $Ez_{t}\varepsilon_{t}=0$, $Es_{t}\varepsilon_{t}=0$. $G\left(s_{t},\gamma ,c\right)$ is the transition function, which is a continuous function with lower bound 0 and upper bound 1. The transition function describes how regimes transit from one to another. In the transition function, $s_{t}$ is the transition variable, which can be one of the random variables in $z_{t}$, or the time trend t, or a linear combination of them. $s_{t}$ represents the cause of regime transition. γ is the smooth transition coefficient, which is positive and represents the degree of smoothness for the regime transition. A smaller γ means a smoother transition. c is the threshold parameter, which describes the time and location of regime transition. The transition function $G\left(s_{t},\gamma ,c\right)$ may take different forms, such as exponential and logistic functions. The corresponding STR models are named Exponential STR (ESTR) and Logistic STR (LSTR). The LSTR model may take the form of LSTR1 or LSTR2 for different forms of the transition function.

#### 2.1.2. Markov Regime-Switching Regression Model

The MRS model was proposed by Hamilton to study the economic cycles [26]. The basic assumption is that the transition among regimes happens randomly. One regime may transit to any possible regimes including itself. The transition process is described by the status transition matrix described as:

(2) $$A=\left[\begin{array}{ccc} a_{11} & \ldots & a_{1k}\\ \vdots & a_{ij} & \vdots \\ a_{k1} & \cdots & a_{kk} \end{array}\right],\;i,j=1,\;2,\;\ldots ,\;k.$$

In the matrix $a_{ij}$ represents the probability of transition from regime i to regime j, and k represents the number of regimes. The MRS model with n independent variables and k regimes is described as:

(3) $$\begin{array}{c} y_{1t}=a_{11}x_{1}+a_{12}x_{2}+\cdots +a_{1n}x_{n}+u_{1t}\\ y_{2t}=a_{21}x_{1}+a_{22}x_{2}+\cdots +a_{2n}x_{n}+u_{2t}\\ \begin{array}{c} \vdots \\ y_{kt}=a_{k1}x_{1}+a_{k2}x_{2}+\cdots +a_{kn}x_{n}+u_{kt} \end{array} \end{array}.$$

The residual $u_{it}$ is assumed to follow a normal distribution with expectation zero and homoscedasticity. The parameters can be estimated by maximum likelihood method.

### 2.2. Model Specification and Data Description

This paper uses GDP per capita to measure the economic development. The GDP per capita data from 1985 to 2016 is from *China Statistical Yearbook*, adjusted to the 1985 price level. By definition, the carbon productivity is calculated using the GDP divided by the carbon emissions each year. The corresponding carbon emission level in the period is estimated using energy consumption data from *China Energy Statistical Yearbook*. China’s energy structure did not change significantly during the research period as fossil energy is still the absolute pillar of China’s energy supply. Carbon emission level can be approximately estimated by energy conversion method. Different types of fossil energy are converted into standard coal through relevant energy factors, and then carbon emissions are calculated using carbon emission factor of standard coal equivalent [27,28,29]. The carbon emission factor of standard coal equivalent varies according to the differences in technical conditions between countries and regions. In this paper, we use the standard coal equivalent emission factor of 0.67 (i.e., each unit of standard coal equivalent generates 0.67 unit of carbon emission) recommended by Energy Research Institute in National Development and Reform Commission of China [30]. Although such assumption does cause uncertainties in estimating exact carbon emissions, our sensitivity test show that the main results are robust in a reasonable range around 0.67. For reducing heteroscedasticity, we take logarithms of GDP per capita and carbon productivity, and then get the time series of lnPGDP and lnCP.

The goal of this study is to establish a parsimonious model to investigate the relationship between lnCP and lnPGDP. This reduced-form specification excludes variables other than GDP per capita. Indeed, there are many variables which represent endogenous consequences of economic growth and exogenous difference. In our simple model, the variables of endogenous consequences of economic growth are omitted because the objective of this study is to assess both direct and indirect consequences of economic growth. The exogenous factors are omitted thus they enter the time trend t and the residual of the model. We will provide detailed explanation on the time trend, and carefully examine the residual to ensure it fits the model assumption.

## 3. Results

### 3.1. Unit Root Test

To avoid spurious regression, we first test whether the data is stationary using the Augmented Dickey-Fuller test (ADF) and Phillips-Perron test (PP). The result is presented in Table 1. Neither lnPGDP nor lnCP are stationary, but both of their first differences are stationary processes. Therefore, the time series of lnPGDP and lnCP are nonstationary I(1) processes.

### 3.2. Nonlinear Effects Test

The nonlinear effects test checks whether nonlinear regime transition exists between different variables. The Brick-Dechert-Scheinkman (BDS) test which was proposed by Broock et al. [31] is commonly used to test whether stochastic nonlinear effects exist among variables. We first establish a vector autoregression (VAR) model for lnPGDP and lnCP to filer the linear relationship between the two variables, and then run the BDS test on the residuals of the VAR model. If the null hypothesis of iid residuals is rejected, then the variables lnPGDP and lnCP have a nonlinear relationship. The results are summarized in Table 2.

The results show that the null hypothesis of linear effect cannot be rejected if lnPGDP is the dependent variable in the VAR model, but the null hypothesis of linear effect is rejected if lnCP is the dependent variable under the dimension of 3, 4, 5, 6. Thus we conclude that lnPGDP has a nonlinear impact on lnCP, but lnCP does not have a significant nonlinear impact on lnPGDP.

### 3.3. Determining the Number of Thresholds in the Nonlinear Effect

Given that lnPGDP has a nonlinear impact on lnCP, the next step is to verify the characteristics of the nonlinear effect in the context of regime transition. We adopt the Likelihood Ratio test (LR) statistics [32] to test the number of thresholds for the variables in the nonlinear effect. The LR statistics are constructed as follows:

(4) $$LR_{ij}=T\left(\ln\left(det{\hat{\Sigma }}_{i}\right)-\ln\left(det{\hat{\Sigma }}_{j}\right)\right),$$

in which ${\hat{\Sigma }}_{i}$ is the covariance matrix assuming there are i thresholds in the model, and ${\hat{\Sigma }}_{j}$ is the covariance matrix assuming there are j thresholds in the model. We test for the hypothesis of no threshold, one threshold and two thresholds sequentially, and the results are shown in Table 3.

According to the results reported in Table 2, in Case 2 the LR statistics value is 61.4425, thus we reject the null hypothesis of no threshold under the 5% significance level. This indicates that there may exist one or two thresholds in the nonlinear effect of lnPGDP on lnCP. The result in Case 3 further rejects the null hypothesis of one threshold. In summary, lnPGDP has a one-way nonlinear impact on lnCP, and this nonlinear impact can be characterized by two thresholds.

### 3.4. Results of the STR Model

Given the characteristics of the nonlinear impact of lnPGDP on lnCP, we then use STR model to further investigate this nonlinear impact. The first step is to determine the form of a basic linear dynamic model. Following the method in Zhao and Fan [33], we use the 1st–4th order lag terms of lnCP, and the 0th–3rd order lag terms of lnPGDP as the candidates for the linear model. After comparing the sixteen combinations, we finally conclude that in the basic linear dynamic model the best order of lags for lnCP is 2, and lnPGDP does not need to contain any lags (see Table 4).

In this linear model, all the estimated coefficients pass the *t*-test at the significance level of 5%; and the Durbin-Watson (DW) statistics is 2.1867, indicating no autocorrelation among the residuals. The result of estimation is $\ln CP_{t}=0.8063+1.2392\ln CP_{t-1}-0.4226\ln CP_{t-2}+0.0880\ln PGDP_{t}.$ The next step is to determine the transition variable. The results are given in Table 5.

When the transition variable is time Trend, there exists regime transition for the impact of lnPGDP on lnCP under the significance level of 5%. We then test the STR model using Trend as the transition variable. The results are shown in Table 6.

In Table 6, $H_{0}^{4}$, $H_{0}^{3}$, $H_{0}^{2}$ are rejected under the 5% significance level. The p-value for rejecting $H_{0,4}$ is the smallest, thus the STR model should take the form of LSTR2 [34]. Then we use point searching method proposed by Teräsvirta to estimate the initial value of the transition function. The range for $c_{1}$ and $c_{2}$ is set as [1.0000, 29.0000] and the range for γ is set as [0.5000, 15.0000]. In each range we take 50 points with equal distance to their neighboring points, constructing 12,500 combinations of parameters. For each combination of $c_{1}$, $c_{2}$ and γ, we calculate the sum of square for the residuals. The parameter combination with the smallest sum of square for the residuals is set as the initial parameters, as shown in Table 7.

The initial values of $c_{1}$, $c_{2}$ and γ fall within their ranges respectively. This result is required by Teräsvirta for further optimization of these parameters. We then adopt the Newton-Raphson iteration algorithm to maximize the conditional likelihood function to get the estimation for the model parameters. After eliminating insignificant independent variables, the corresponding parameters of the LSTR2 model are presented in Table 8.

The final expression of the LSTR2 model is:

(5) $$\ln CP_{t}=3.1046+1.0748\ln CP_{t-1}-0.5562\ln CP_{t-2}+0.1067\ln PGDP_{t}\;+\left(1.4862-1.3244\ln CP_{t-1}+0.6782\ln CP_{t-2}+0.4652\ln PGDP_{t}\right)\times G\left(s_{t},\gamma ,c\right),$$

in which $G\left(s_{t},\gamma ,c\right)={\left[1+\exp\left[-11.8443\left(t-10.9018\right)\left(t-26.5203\right)\right]\right]}^{-1}$.

The model divides the impact of $\ln PGDP_{t}$ on $\ln CP_{t}$ into two parts. One part is $\left(3.1046+1.0748\ln CP_{t-1}-0.5562\ln CP_{t-2}+0.1067\ln PGDP_{t}\right)$, which is linear. The other part is $\left(1.4862-1.3244\ln CP_{t-1}+0.6782\ln CP_{t-2}+0.4652\ln PGDP_{t}\right)\times G\left(s_{t},\gamma ,c\right)$, which is nonlinear. According to the definition of the LSTR2 model, the influence of $\ln PGDP_{t}$ on $\ln CP_{t}$ is converted by $G\left(s_{t},\gamma ,c\right)$. When the transition function $G\left(s_{t},\gamma ,c\right)$ is close to one, the influence of $\ln PGDP_{t}$ on $\ln CP_{t}$ is the overlap of the two parts. The formula is $\left(4.5908-0.2496\ln CP_{t-1}+0.1220\ln CP_{t-2}+0.5719\ln PGDP_{t}\right)$. In this situation, the intensity of $\ln PGDP_{t}$’s influence on $\ln CP_{t}$ is 0.5719. When $G\left(s_{t},\gamma ,c\right)$ is close to zero, the influence of $\ln PGDP_{t}$ on $\ln CP_{t}$ is just the linear part. In this situation, the intensity of $\ln PGDP_{t}$’s influence on $\ln CP_{t}$ is 0.1067. After comparison, we found the carbon productivity grows faster as the GDP per capita increases in the nonlinear part, thus we label this regime as the high regime. On the other hand, when $G\left(s_{t},\gamma ,c\right)$ approaches zero, the carbon productivity grows slower as the GDP per capita increases, hence we label this regime as the low regime.

**Figure 1:**
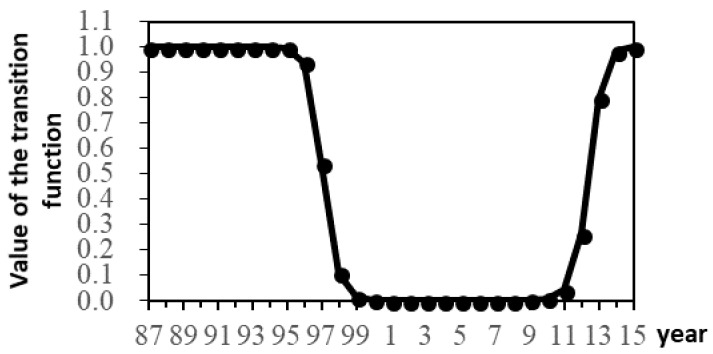
Changes of the transition function.

In the LSTR2 model, $c_{1}$ and $c_{2}$ are the threshold parameters, which describe the time of regime transition. When transition variable is less than $c_{1}$ or when transition variable is more than $c_{2}$, the transition function $G\left(s_{t},\gamma ,c\right)$ is close to one. When transition variable is between $c_{1}$ and $c_{2}$, $G\left(s_{t},\gamma ,c\right)$ is close to zero. We depict the transition dynamics in Figure 1, where we observe that the impact of $\ln PGDP_{t}$ on $\ln CP_{t}$ experiences a transition from the high regime to the low regime, and back to the high regime again. There exist an obvious two-regime transition pattern. More specifically, the economic-growth-to-carbon-productivity relationship was in high regime from 1987 to 1998, and was in low regime from 1999 to 2010, and turned to high regime again from 2011 to 2015 in Hubei Province. We conjecture that the underlying reasons for high and low regimes are variations in economic structure and technology upgrade. The first high regime in Hubei may be the result of a combination of a weak secondary industry and a growing tertiary industry. The low regime coincided with the period of massive infrastructure investment. The second high regime may be driven by technology upgrade. We will provide further comments on these underlying reasons in the discussion section. The value of the smooth transition coefficient γ is large, indicating a fast transition. In Figure 1, the transitions between high regime and low regime finish within about three years.

At last we verify whether the residuals of the LSTR2 model satisfies the model assumption. The results show that the residuals have homoscedasticity of variance, no serial correlation and subject to normal distributions. Therefore, the LSTR2 model describing the nonlinear relationship of $\ln PGDP_{t}$ to $\ln CP_{t}$ is robust.

The LSTR2 model outperforms simple linear models in fitting the data. Table 9 compares the fitness and the standard deviation of residuals for the LSTR2 model and the linear model. LSTR2 model has higher $R^{2}$ and significantly smaller standard deviation of residuals. Hence the LSTR2 model fits the data better than the linear model.

### 3.5. Results of the MRS Model

According to the results of the STR model, there are two regimes in the nonlinear relationship between economic growth and carbon productivity in Hubei, and the transitions are finished in a short time. The result fits the assumption required by the MRS model that transitions happen instantly. Hence it is appropriate to apply the two-regime MRS model to investigate the probability of regime transitions that can be used to predict future status. The transition probability matrix is calculated and shown in Table 10.

If the impact of $\ln PGDP_{t}$ on $\ln CP_{t}$ is in the low regime in a certain period, then in the next period the probability of staying in the low regime is 0.8009, and the probability of jumping to the high regime is 0.1991. Meanwhile, if the impact of $\ln PGDP_{t}$ on $\ln CP_{t}$ is in the high regime, then in the next period the probability of staying in the high regime is 0.9523, and the probability of jumping to the low regime is 0.0477. The high regime is more sustainable than the low regime.

We derive the expected duration of the high regime and the low regime using the formula $1/\left(1-p_{ii}\right)$. The variable $p_{ii}$ represents the probability of staying in the previous regime. For Hubei province, the expected duration of staying in the low regime is 5 periods, and the expected duration of staying in the high regime is 20 periods. The high regime is more stable.

## 4. Discussion

The first main result of our study is that economic growth has a constantly positive impact on increasing carbon productivity. In other words, carbon productivity increases monotonically as GDP per capita increases. This is a desirable characteristic for developing economies. However, many previous studies such as Shafik and Bandyopadhyay [35], and Wang et al. [14] find the characteristic that carbon emissions per capita also increase monotonically as GDP per capita grows. In fact, there is no contradiction between the two seemingly conflicting results because the dependent variables are different. It is possible that both carbon productivity and carbon emission per capita rise as the GDP per capita grows. An increase of absolute carbon emissions leads to higher carbon emissions per capita for a stable population, and can also lead to higher carbon productivity if the GDP grows even faster. In Hubei Province the GDP growth rate reached 13.8% in 2011, which is very high. This may partially explain the positive relationship between economic growth and carbon productivity in Hubei. Therefore, enhancing carbon productivity is a weaker objective for policy makers than lowering carbon emission per capita in regions with fast economic growth and a stable population. We need to be cautious about the result if otherwise slowly-growing economies are analyzed.

The positive impact of economic growth on carbon productivity is very similar to the positive effect of economic growth on reducing water pollution and improving urban sanitation [35]. The common underlying driving force of these similar results may be internalizing the cost of externalities of undesired outputs. In the literature such positive effects are usually reported for local pollutants. One explanation in environmental economics theory is that local pollutants’ negative impacts are easier to be internalized in a region than globally externalized pollutants like CO_2_, thus strict local environmental policies can be effectively made. In China the carbon productivity has entered the KPI of provincial governments since 2014, thus the external social cost of carbon emissions is internalized to some extent. It may partially explain why carbon productivity is positively impacted by economic growth in Hubei.

Another result is the characteristic of transition between two regimes. From a perspective other than the EKC theory, we draw the time path of changes in carbon productivity. For the case of Hubei, if the time path of carbon productivity is put in the context of its economic development history, we may acquire a better understanding of its underlying patterns. Before 1998, the economic growth in Hubei lagged behind the coastal areas of China, and its previously strong secondary industry could not get enough investment thus was declining. The economic growth mainly relied on less capital-intensive tertiary industry, which emit less carbon dioxide. Hence the carbon productivity increased in the high regime. Between 1999 and 2010, a boost in economics happened with massive infrastructure construction like the Three Gorges project. The high-emission industries and urbanization developed fast thus the carbon productivity increased in the low regime. After 2010 the provincial government began to transit to low-carbon economy. The secondary industry was upgrading its production technology. The carbon exchange market was established. More policies and regulations encouraging low-carbon development were implemented. Then we observe that the carbon productivity grew in the high regime again.

It is the high regime that is more stable and lasts for longer time according to the results of MRS model. This is a reasonable result. In Hubei the low regime is associated to the developing period of massive infrastructure construction and extensive economic growth, which is not sustainable for a long period. The high regime is triggered by technology upgrade that may last for longer time. The transition between regimes happens very fast, which coincides with Zhao and Fan [33]. The fast transition makes the MRS model more applicable, and may be an indicator of local government’s efficiency in designing and executing policies.

It should be noted that a slight increase in carbon productivity is not enough to control the absolute carbon emission level. Although economic growth enhances the carbon productivity, the government should not stop at focusing on economic growth only. To curb the absolute carbon emissions is a challenging task and every effort should be exerted.

## 5. Conclusions

This paper investigates the relationship between economic growth and carbon productivity in Hubei by combining smooth transition regression model and Markov regime-switching regression model. The results indicate that economic growth in general has a positive impact on increasing carbon productivity, and this positive impact is highly nonlinear across different time periods. There exist two regimes, namely, a high regime and a low regime, in the relationship between economic growth and carbon productivity. The transition between regimes is two-way and fast. The high regime, in which economic growth has a stronger positive impact on enhancing carbon productivity, is expected to last four times longer than the low regime. It is more probable for a low regime to transit to a high regime. Once the relationship of carbon productivity and economic growth enters the high regime status, it becomes relatively stable. However, simply pursuing higher carbon productivity is not enough in curbing the absolute carbon emissions, thus various means should be taken to control the carbon emissions.

Based on our findings, we propose the following policy implications. As economic growth always positively influences carbon productivity, in order to achieve higher carbon productivity, it is helpful to encourage stronger economic development. The fast-developing economies do not need to worry too much that the goals of economic growth and carbon productivity may conflict with each other. Since in the high regime economic growth has a stronger positive impact on carbon productivity, it is ideal to enter and stay in the high regime for enhancing carbon productivity. According to the case of Hubei, a high regime is associated with technology upgrades and economic structure optimization, hence to accelerate the optimization and upgrade of industrial structure, to transform the coal-based energy structure by developing clean energy, to actively cultivate the tertiary industry, and to promote the development of recycling and low carbon technology should be advocated. Policies in these aspects would lay a solid foundation for long-term stable development in a high regime.

The results obtained can be applied in other areas with similar energy structure and economic context. However, if significant changes in energy structure or disruptions in economic development occur, there may be more regimes and complex dynamics. In future studies, the model can be extended to incorporate detailed factors influencing carbon productivity to support more specific policy implications. Meanwhile, the results in different regions can be compared to derive managerial insights for policy makers.

## Figures and Tables

**Table 1:** Unit root test results.

Variables	Test Form	ADF Test		PP Test		Result
		*t*-Statistic	*p*-Value	*t*-Statistic	*p*-Value	
lnPGDP	(c,t)	−2.5810	0.2907	−3.1469	0.1142	I(1)
ΔlnPGDP	(c,0)	−3.0565	0.0464	3.2762	0.0355	I(0)
lnCP	(c,t)	−2.0919	0.5286	−2.0025	0.5764	I(1)
ΔlnCP	(c,0)	−3.5907	0.0123	−3.6322	0.0114	I(0)

Note: crepresents intercept term; t represents trend item.

**Table 2:** BDS nonlinear effect test result based on VAR model residuals.

Dimension	ResidlnPGDP		ResidlnCP	
	*Z*-Statistic	*p*-Value	*Z*-Statistic	*p*-Value
2	0.9433	0.3455	−1.6718	0.0946
3	1.3632	0.1728	−3.8568	0.0001
4	1.6694	0.0950	−2.6737	0.0075
5	1.5484	0.1215	−2.6741	0.0075
6	1.0559	0.2910	−2.3944	0.0166

Note: (1) The optimal delay order of VAR model is 2, which is selected by Akaike Information Criterion (AIC) information criterion, similarly hereinafter. (2) ResidlnPGDP is a residual sequence in the VAR model, in which the lnPGDP is used as the dependent variable, residlnCP is a sequence of residual errors obtained by using lnCP as the dependent variable in the VAR model.

**Table 3:** Likelihood ratiotest for nonlinear effect.

Three Cases	Illustration	LR-Statistic	*p*-Value
Case 1: *i* = 1, *j* = 0	$H_{0}$: no threshold $H_{1}$: one threshold	22.2057	0.2630
Case 2: *i* = 2, *j* = 0	$H_{0}$: no threshold $H_{1}$: two thresholds	61.4425	0.0140
Case 3: *i* = 2, *j* = 1	$H_{0}$: one threshold $H_{1}$: two thresholds	39.2369	0.0150

**Table 4:** Result of base linear dynamic model.

Variable	Coefficient	*t*-Statistic	*p*-Value
CONST	0.8064	1.8369	0.0781
$\ln CP_{t-1}$	1.2392	6.9013	0.0000
$\ln CP_{t-2}$	−0.4226	−2.3362	0.0278
$\ln PGDP_{t}$	0.0880	2.1241	0.0437
Adjusted *R*^2^		0.9880	
Durbin-Watson Statistics		2.1867	

**Table 5:** Result of nonlinear effect.

**Transition variable**	$\ln CP_{t-1}$	$\ln CP_{t-2}$	lnCP	Trend
***p*-value**	NaN	NaN	NaN	0.0134

Note: (1) The null hypothesis is $H_{0}^{0}$: There is no nonlinear effect. (2) The *p*-value corresponds to the F statistic. (3) NaN indicates that the inverse matrix does not exist thus cannot be calculated.

**Table 6:** Results of smooth transition regressionmodel form.

**The null hypothesis**	$H_{0}^{4}$	$H_{0}^{3}$	$H_{0}^{2}$	Model form
**Transition variable:** Trend	0.0238	0.0248	0.0446	LSTR2

Note: (1) the numbers are the *p*-value corresponding to the F statistic; (2) $H_{0}^{4}$, $H_{0}^{3}$, $H_{0}^{2}$ are the null hypotheses corresponding to the Teräsvirta solution, respectively.

**Table 7:** Initial estimate results of $c_{1}$, $c_{2}$ and γ.

Variable	Value Range	The Initial Value
$c_{1}$	[1.0000,29.0000]	10.7143
$c_{2}$	[1.0000,29.0000]	26.7143
γ	[0.5000,15.0000]	11.3634

**Table 8:** LSTR2 model estimation results.

Variable	Coefficient	*t*-Statistic	*p*-Value
Linear part			
CONST	3.1046	3.1262	0.0057
$\ln CP_{t-1}$	1.0748	5.0812	0.0001
$\ln CP_{t-2}$	−0.5562	−3.2698	0.0043
$\ln PGDP_{t}$	0.1067	3.0765	0.0065
Non-linear part			
CONST	1.4862	0.9332	0.3631
$\ln CP_{t-1}$	−1.3244	−0.0396	0.0071
$\ln CP_{t-2}$	0.6782	2.6105	0.0177
$\ln PGDP_{t}$	0.4652	2.8053	0.0117
γ	11.8443	2.2908	0.0343
$c_{1}$	10.9018	30.4057	0.0000
$c_{2}$	26.5903	91.3949	0.0000
Adjusted $R^{2}$		0.9974	
S.D. of residuals		0.0262	

**Table 9:** Comparison between linear model and nonlinear model.

Model Form	$R^{2}$	Adj. *R*^2^	SD of Resid.
Linear	0.9893	0.9880	0.2106
LSTR2	0.9974	0.9974	0.0262

**Table 10:** Transition probability matrix of MRS model.

	*j* Status	Low-Regime	High-Regime
*i* Status			
Low-regime		0.8009	0.1991
High-regime		0.0477	0.9523
